# Poorer Integration of Local Orientation Information Occurs in Students With High Schizotypal Personality Traits

**DOI:** 10.3389/fpsyt.2018.00518

**Published:** 2018-10-24

**Authors:** Kirsten R. Panton, Johanna C. Badcock, J. Edwin Dickinson, David R. Badcock

**Affiliations:** ^1^Human Vision Laboratory, School of Psychological Science, University of Western Australia, Perth, WA, Australia; ^2^Division of Psychiatry, Faculty of Health and Medical Sciences, Centre for Clinical Research in Neuropsychiatry, University of Western Australia, Perth, WA, Australia

**Keywords:** schizotypy, perceptual organization, visual integration, schizophrenia, global procesisng, local processing

## Abstract

Contour integration is impaired in schizophrenia patients, even at the first episode, but little is known about visual integration abilities prior to illness onset. To examine this issue, we compared undergraduate students high and low in schizotypal personality traits, reflecting putative liability to psychosis, on two psychophysical tasks assessing local and global stages of the integration process. The Radial Frequency Jittered Orientation Tolerance (RFJOT) task measures tolerance to orientation noise at the local signal level, when judging global stimulus orientation, whilst the Radial Frequency Integration Task (RFIT) measures the ability to globally integrate the local signals that have been extracted during shape discrimination. Positive schizotypy was assessed with the Perceptual Aberration (PAb) scale from the Wisconsin Schizotypy Scales-Brief. On the RFJOT task, the High PAb group (*n* = 55) tolerated statistically significantly less noise (*d* = −0.494) and had a lower proportion of correct responses (*d* = −0.461) than the Low PAb group (*n* = 77). For the RFIT there was no statistically significant difference in integration abilities between the High and Low PAb groups. High and Low PAb groups also differed on other positive and disorganized (but not negative) schizotypy traits, hence poorer performance on the RFJOT may not be solely related to unusual perceptual experiences. These findings suggest that difficulties with local noise tolerance but not global integration occur in healthy young adults with high levels of schizotypal personality traits, and may be worth investigating as a marker of risk for schizophrenia.

## Introduction

Visual perceptual disturbances are common in schizophrenia (1, 2) and in schizotypy (3, 4)—a cluster of personality traits reflecting a putative liability to schizophrenia-spectrum disorders in the general population (5, 6). In particular, difficulties in perceptual organization (PO)—the integration of visual information into coherent patterns—have been well-documented in both groups (7–11). It has also been suggested that abnormal PO may be relatively common in neurodevelopmental conditions, such as schizophrenia spectrum (2) and autism spectrum disorders (12, 13), though the exact nature of these difficulties is still unclear.

**Figure 1:**
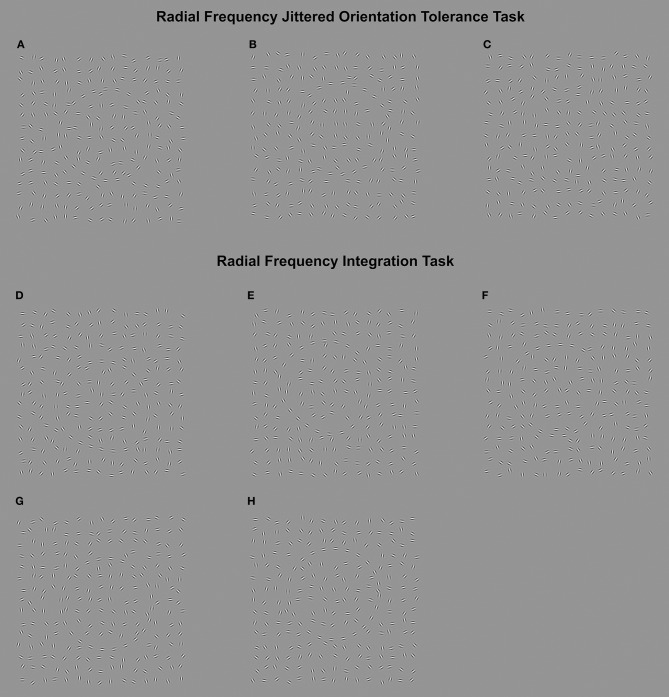
Upper: (Radial Frequency Jittered Orientation Tolerance task). **A–C** in the upper part of the figure represent sample items from the Radial Frequency Jittered Orientation Tolerance (RFJOT) task. The participant is required to indicate whether the RF3 is pointed to the left (examples **A**,**C** point left) or right (example **B**) The amount of orientation jitter increases from example **(A–C)**, showing 0°, 17°, and 27° of jitter, respectively. Participants are expected to perform at close to ceiling for zero jitter levels **(A)**, with reduced accuracy as jitter is increased up to 27° jitter **(C)**. The jitter level presented in stimulus **(B)** (17°) is the approximate average threshold across all participants. Lower: (Radial Frequency Integration Task). The Radial Frequency Integration Task (RFIT) measures an individuals' amplitude threshold to discriminate an RF3 **(D–G)** from a circle **(H)** when 1 cycle **(D)**, 2 cycles **(E)** and 3 cycles **(F,G)** are present. **D–F** represent the RF patterns at 2.5 times the average threshold across participants, whereas **(G)** shows an RF3 at the average threshold for 3 cycles.

In the schizophrenia literature, there has been a strong research interest in *contour integration* as an index of PO, particularly using the Jittered Orientation Visual Integration [JOVI; (14)] task, which measures an individual's ability to tolerate orientation noise at the local signal level when evaluating the shape of a global contour formed from these elements. In this task, the stimulus is a field of randomly oriented (but relatively evenly spaced) Gabor patches, containing the target shape (commonly an egg-shape). The target shape is made up of regularly sampled Gabor elements, which becomes visible by aligning the Gabor patches with the boundary of the shape (see Figures 1A–C). The participants' task is to decide which direction the target shape is pointing when elements on the contour are randomly changed in orientation by varied amounts (which increases task difficulty). Threshold performance on this task reflects the amount of orientation noise that can be tolerated when making these judgments. Although this task has received strong research interest, and consistently revealed poorer performance in schizophrenia patients [e.g., (14–17)], as early as the first psychotic episode (18), much less is known about these particular integration abilities in healthy individuals with high levels of schizotypy, who are at increased risk for schizophrenia.

One previous study, using a task with similar stimulus and processing requirements as the JOVI, compared high and low schizotypal groups [individual's scoring in the top and bottom 20% on cognitive-perceptual, interpersonal and disorganized traits on the Schizotypal Personality Questionnaire; (19)], but found no differences between these groups on integration abilities. In contrast, disorganized schizotypy, assessed with the Thought Disorder Index (20) was associated with poorer integration abilities (21). Consequently, it is difficult to know the true interaction between schizotypal traits and integration abilities. More recently, Carter and colleagues have argued that visual integration deficits are closely related to the positive (e.g., delusions, hallucinations) symptoms of schizophrenia, independent of diagnosis (22); though visual motion, rather than contour integration was examined, which primarily activates dorsal rather than ventral cortical pathways (23), and the relationship with positive, disorganized and negative schizotypy traits was not investigated.

The JOVI task involves both local and global aspects of contour integration (24), which may independently influence PO. The task requires the orientation of local elements to be extracted to form a smooth, albeit sampled, shape and then a judgement must be made about a global property of that shape which is only available after the shape is formed; whether it points left or right. Poor performance could arise from either impaired performance in the ability to distinguish the orientation samples on the path from the noise (*local uncertainty*), or a degraded representation of the shape due to the incorporation of orientation noise on the sampled path (*global uncertainty*). Since the global task is very simple and common across conditions in which local element noise is varied, variation in orientation noise tolerance is likely to reflect local element extraction properties. Alternatively, poor performance on the JOVI could arise from global integration difficulties; however, local and global processes are not easily distinguished with current versions of this task.

Here we use the Radial Frequency Integration Task (RFIT), to assess the efficiency of *global* integration of local shape information during shape discrimination. Radial Frequency (RF) patterns are distorted circles created by the regular sinusoidal modulation of the radius of the circle around it's perimeter (25). The RFIT measures the threshold depth of sinusoidal distortion of the RF contour needed to discriminate a contour from a circle. The threshold is measured for patterns with different proportions of the perimeter undergoing distortion [Figures 1D–H; (26–31)]. Sensitivity to local orientation signals determines the minimum detectable amplitude of modulation (30) and as more local information is provided, thresholds decrease. This relationship is represented by the *slope* of the line indicating improvement in performance, and provides an index of global integration. If the slope is steeper than can be predicted simply by having more local distortion signals to detect (i.e., by probability summation), then there is evidence for global contour integration (32–34).

Complementing the RFIT, we created a modified version of the JOVI task–the Radial Frequency Jittered Orientation Task (RFJOT). Importantly, the stimuli in the tasks used here—RF patterns—are readily able to differentiate local and global visual processes contributing to PO performance (35). For both the RFIT and RFJOT an RF pattern where three cycles of modulation would complete the 360° contour (an RF3) were used, as these have been shown to drive global integration in the visual system (31–33). Previous work has shown atypically enhanced global integration in “healthy” adults high in autistic-like traits (26). To date, however, the RFJOT and RFIT has not been used to examine contour integration abilities in a schizotypy sample.

Using both the RFIT and the RFJOT allows for more precise determination of the level of processing difficulties that may be associated with schizotypal traits. If the RFIT produces higher overall thresholds, this would indicate a problem with the local level of element processing while a different rate of improvement with additional cycles of modulation on the contour would indicate a difference in global contour integration. The RFJOT determines the tolerance to local contour features (element orientation) but does not place a stringent requirement on the global integration abilities. Using the two tasks together allows measurement of the strength of global integration of contour information (RFIT, integration slope)^1^ and tolerance of local orientation noise (RFJOT threshold).

In sum, our aim was to investigate contour integration abilities in healthy young adults high and low in schizotypy traits using these two psychophysical tasks: 1) the Radial Frequency Jittered Orientation Tolerance (RFJOT) task and 2) the RFIT, to assess local and global visual integration, respectively. Building on prior research, we chose to focus on positive schizotypy, comparing visual integration abilities in healthy individuals with high and low scores on the Perceptual Aberrations (PAb) scale (36). Based on previous evidence [e.g., (14, 18, 21, 22)] we predicted that, compared to the Low PAb group, the High PAb group would exhibit poorer tolerance to local noise (lower thresholds and lower proportion of correct responses on the RFJOT task) and reduced global integration efficiency (shallower slope values on the RFIT). In addition, in order to address the issue of specificity, the influence of other positive, disorganization and negative schizotypy traits was also explored.

## Methods

### Participants

#### Screening and recruitment

Two-thousand one-hundred and ninety-eight undergraduates from the University of Western Australia completed the PAb (see Table S2 for comparison with previous studies) and Social Anhedonia subscales from the Wisconsin Schizotypy Scales-Brief (36) and the Cognitive Disorganization subscale from the short-form Oxford-Liverpool Inventory of Feelings and Experiences (37). Schizotypy questions were interspersed with items from the Infrequency Scale (38) to identify inconsistent or careless responding (1.7% of respondents met this criteria). Participants with High (above 90th percentile, PAb score ≥ 3) and Low (below 50th percentile, PAb score = 0) scores on the PAb scale^2^ were randomly sampled and invited to participate in further testing (outlined below).

#### Clinical assessment and exclusions

Participants were excluded if they reported a past or current diagnosis and/or treatment for a psychotic illness (*n* = 1); screened positive for the presence of psychotic illness on individual interview (41, 42) (*n* = 0)^3^, had a positive history of a neurological disorder (*n* = 1), substance abuse (*n* = 5), poor visual acuity (lower than 20/32, *n* = 0) or poor fluency in English (*n* = 0).

#### Sample characteristics

Fifty-five participants were recruited in the High PAb group (scores ≥3) and 77 participants in the Low PAb group (scores of 0). The sample comprised predominantly young adults (*M*_*age*_ = 19.57, *SD*_*age*_ = 2.95, 17–31 years; 68% female).

### Psychometric measures

#### Schizotypal traits

The Wisconsin Schizotypy Scale-Brief (36) and short-form Oxford-Liverpool Inventory of Feelings and Experiences (37) are reliable and valid self-report scales measuring different aspects of schizotypy (43–45). The Wisconsin Schizotypy Scale-Brief measures positive (PAb and Magical Ideation) and negative (Physical and Social Anhedonia) traits. The Cognitive Disorganization scale from the short-form Oxford-Liverpool Inventory of Feelings and Experiences measures disorganized features of schizotypy.

#### Autism-related traits

The Autism-Spectrum Quotient (AQ) is a self-report measure of autistic-like traits (46), with higher AQ scores indicating more autistic-like behavior. AQ traits were included as a potential confounding factor, associated with better global integration on the RFIT (26).

### Measures of local and global visual integration

#### Radial frequency (RF) patterns

RF patterns are closed-contour shapes generated by sinusoidal modulation of the radius of a circle as a function of polar angle (25),

(1) $$\begin{array}{l} R\left(\theta \right)=R_{0}\left(1+A.\sin\left(\omega \theta +\phi \right)\right) \end{array}$$

where θ is the angle made with the x-axis, *R*_0_ is the mean radius of the modulated circle, *A* is the amplitude of modulation, ω is the frequency of modulated cycles in 360° (ω = 3, denoted RF3) and φ is the angular phase of the shape.

For both tasks described below, a regularly-sampled RF3 pattern (1, 2, or 3 cycle) or circle is displayed amongst noise elements. The orientation of the jittered elements of the RF pattern at each location around the contour varies from the local tangent to a circle, with the same center, and is given by Dickinson et al. (47):

(2) $$\begin{array}{l} \alpha \left(\theta \right)=\tan^{-1}\left(\frac{A\omega \text{ cos }\left(\omega \theta +\varphi \right)}{\frac{R\left(\theta \right)}{R_{0}}}\right) \end{array}$$

where α(θ) is the local Gabor orientation at a specific polar angle around the pattern and other parameters are define as for Equation 1. Both the target contour and noise elements were made of Gabor patches with a grating spatial frequency centered on 4 c/deg and in cosine phase so a bright bar was at the center of the pattern. The target pattern (RF or circle) contained 24 evenly spaced (1.25° separation) Gabor patches with a radius of 3.78°. There were 225 noise elements, which were placed within a 15 × 15 grid, subtending 17.5° visual angle.

#### RF jittered orientation tolerance (RFJOT) task

The RFJOT task (adapted from the JOVI) was used to measure an individuals' tolerance to orientation noise at the local signal level when evaluating a global percept. The target shape was a Radial Frequency (RF) pattern which is a closed-contour shape generated by sinusoidal modulation of the radius of a circle as a function of polar angle (25) (see equation in 1). On each trial, participants had to decide if the corner of the target RF3 (a triangular shape when *A* = 0.025 in Equation 1) was facing leftward or rightward (leftward 50% of the time). The elements on the contour were jittered in orientation by varied amounts, such that the coherence and detectability of the contour declines (see Figures 1A–C). Jitter levels similar to Silverstein et al's (14). Study 1 (±0, ±7, ±11, ±15, ±19, ±23, ±27 degrees of jitter) were used in order to sample a range of accuracy levels (50–100%). The amount of orientation jitter required to reduce performance to 75% (threshold^4^) served as our primary measure of performance. As in Silverstein et al. (14) the proportion of correct responses combined across the jitter levels was calculated, providing a secondary measure of performance (see Supplementary Materials for details).

Fourteen practice trials (2 trials per jitter level) were administered prior to the task. Twenty test trials (140 trials in total) at each jitter level were presented, which were randomly interleaved. Participants' pressed the left or right button on a button-box to indicate the direction pointed by the target. There was no time limit to make a response, however, reaction time was still recorded to 100 μs resolution. Reaction time data will not be analyzed here. Participants were informed to respond as quickly and accurately as possible. No feedback was provided.

#### RF integration task (RFIT)

The RFIT assessed the ability to globally integrate information around a regularly sampled contour (Figures 1D–H). The target was either a sampled RF3 pattern or a sampled circle, displayed amongst noise presented in a 2-interval forced-choice procedure. The participants' task was to indicate whether the RF3 appeared in the first interval (left-button press) or second interval (right-button press). The amplitude (*A*) of shape distortion required to discriminate an RF3 from a circle was measured, as more modulation cycles (from here on referred to as *corners*), are added to an otherwise circular contour (Figures 1D–H). Integration ability was indicated by the index of the power function (*B*, see *equation S2*, Supplementary Materials), describing the rate of threshold (minimum amplitude of shape distortion) decrease with increasing number of corners. Global integration is evident if *B* (referring to the slope of performance) is statistically significantly steeper than probability summation. Probability summation refers to the increased probability of detecting a single corner as the number of corners presented increases, and assumes a purely local processing of the contour (32, 33, 48, 49).

The threshold was obtained using Psi, an adaptive psychophysical procedure implemented through the Palamedes toolbox in Matlab [see Chapter 4, (50)]. The presentation of the test (RF3) and reference (circle) patterns were randomized and presented for 160 ms each, with a 500 ms inter-stimulus interval. The orientation of the test pattern varied between trials to prevent the participant from knowing the deformation location prior to the trial. Before the test trials, each individual completed approximately 10 practice trials when all three cycles were present to familiarize participants with the task. Experimental trials commenced with an amplitude (A in Equation 1) of 0.1 and one-hundred test trials were presented in each run for each number of corners. Participants had an unlimited viewing time, but were asked to respond as quickly as possible after the presentation of the stimulus. No feedback was provided.

### Additional tests

Visual acuity can influence some aspects of contour integration (17), so was assessed with a LogMAR acuity chart (using the line scoring method, (51)). Lower scores indicate poorer visual acuity. General cognitive ability was estimated with a tablet-based Digit Symbol Coding test (NeuroCog Trials, (52)) with scores indicating the number of items correctly identified in 90s. Handedness was assessed with the Edinburgh Handedness Inventory (http://zhanglab.wikidot.com/handedness), scores ranged from +1 (pure right handed) to −1 (pure left-handed).

### General procedure

The PAb, Social Anhedonia, Cognitive Disorganization and AQ scales were voluntarily completed in supervised groups in an initial screening session, following normal laboratory classes. Individuals meeting the inclusion criteria on the PAb scale were invited to take part in further testing, in a separate session. All schizotypy scales and the AQ scale were then re-administered, prior to the RFJOT and RFIT, to determine test-retest reliability. Other additional tests were conducted prior to the visual measures. Another subset of PO tasks was also presented, which were part of related experiments to be reported separately. Total testing time was approximately 2.5 h (including rest breaks), and participants received course credits for their participation. The study was approved by the Human Research Ethics Committee at the University of Western Australia. All participants provided informed, written consent and no financial incentives were offered.

### Data analysis

GraphPad Prism Version 7 and IBM SPSS Statistics Version 22 were used to analyse the data. For the RFJOT, individuals with a total proportion correct score < 50% were planned to be removed [as Silverstein et al. (14)], however, none met this criteria. Additionally, individuals performing below 75% accuracy on the 0 jitter level were excluded (High PAb: *n* = 2, Low PAb: *n* = 1), as this indicates that the participant was having difficulty seeing the RF3 stimulus. For the RFIT, the distribution of the integration slopes and probability summation slope estimates were skewed, consequently log-transformed values were used for the analyses reported below (the anti-logged data is presented in figures). Additionally, one outlier (>3SD mean) was detected for the integration index, however, removing this datum did not alter the outcome, and the distribution remained normal, so this datum stayed in the analyses. R^2^ (a goodness of fit statistic) indicated good fits for both the RFJOT (*R*^2^ = 0.875, 95% CI ± 0.021) and RFIT (*R*^2^ = 0.936, 95% CI ± 0.023)^5^.

Spearman's correlations indicated good or acceptable test-retest reliability for schizotypy and AQ scores: PAb = 0.76, Cognitive Disorganization = 0.84, Social Anhedonia = 0.74 and *AQ* = 0.87. Original screening scores were used for the High and Low PAb group allocations reported below. Individuals who scored 3 or more on the Infrequency Scale were excluded from the final sample (High PAb group: *n* = 1). Outliers (> ± 3SD) for age (High PAb: *n* = 1, Low PAb: *n* = 2) were excluded prior to analysis.

Effects sizes for the ANOVA's are represented by partial eta squared (η^2^) and Cohen's d for *t*-tests. Effect sizes are considered small when η^2^ = 0.01/d = 0.20 or approach that value, medium when η^2^ = 0.06/d = 0.50 or approaching that value, and large when approaching η^2^ = 0.14/d = 0.80 (53).

## Results

### Descriptive statistics

There were no statistically significant differences between High and Low PAb groups in age, DSC, handedness or acuity (see Table 1). Chi-squared tests also showed no statistically significant differences in sex distribution between the High and Low PAb groups, χ^2^ = 0.290, *p* = 0.590. Statistically significant group differences were found in (some) other schizotypy traits and for total AQ scores, though effect sizes (Table 1) and score distributions (Figure S2) show the large group overlap on the majority of these measures (other than the PAb traits).

**Table 1:** Participant characteristics in High and Low PAb groups.

	**High PAb (*n* = 55)**	**Low PAb (*n* = 77)**	***t***	***p***	***d***
Perceptual aberration	4.95 (2.30)	0 (0.00)	18.44	<0.001^*^	3.03
Cognitive disorganization	7.27 (2.64)	5.09 (3.15)	4.19	<0.001^*^	0.75
Magical ideation	4.35 (2.70)	1.45 (1.85)	7.33	<0.001^*^	1.25
Social anhedonia	3.47 (3.34)	2.54 (2.84)	1.72	0.09	0.30
Physical anhedonia	2.05 (1.86)	2.21 (2.05)	−0.49	0.66	−0.08
Total autism quotient	20.00 (7.00)	16.14 (6.40)	3.28	0.001^*^	0.57
Acuity	−0.09 (0.08)	−0.11 (0.08)	1.35	0.09	0.31
Digit symbol coding	59.62 (9.36)	60.73 (9.71)	−0.56	0.51	−0.12
Age	19.64 (3.09)	19.53 (2.79)	0.23	0.84	0.04
Handedness^∧^	0.73 (0.39)	0.66 (0.52)	2113.50	0.99	−0.02

* *p is significant at 0.05 level*.

∧ *Mann-Whitney U test was used, and the effect size is represented by Z*.

Correlations between DSC, handedness, acuity and AQ scores with RFJOT thresholds and RFIT integration slopes were explored to identify potential covariates (Table S3). Correlations larger than 0.3 were used to identify potential covariates (54). All correlations were low, and none exceeded this criterion (54). Given *a priori* evidence (17, 26), analyses of visual integration data were repeated with and without AQ and acuity as covariates. The pattern of results was unchanged, hence for brevity only the latter are presented.

### RFJOT

Preliminary inspection of performance at 0° jitter level indicated no statistically significant difference in the proportion of correct responses between High PAb (*M* = 0.972, *SD* = 0.090) and Low PAb (*M* = 0.987, *SD* = 0.039) groups [*t*_(126)_ = −0.232, *p* = 0.817, *d* = −0.041], indicating that all participants displayed adequate attention to the task.

**Figure 2:**
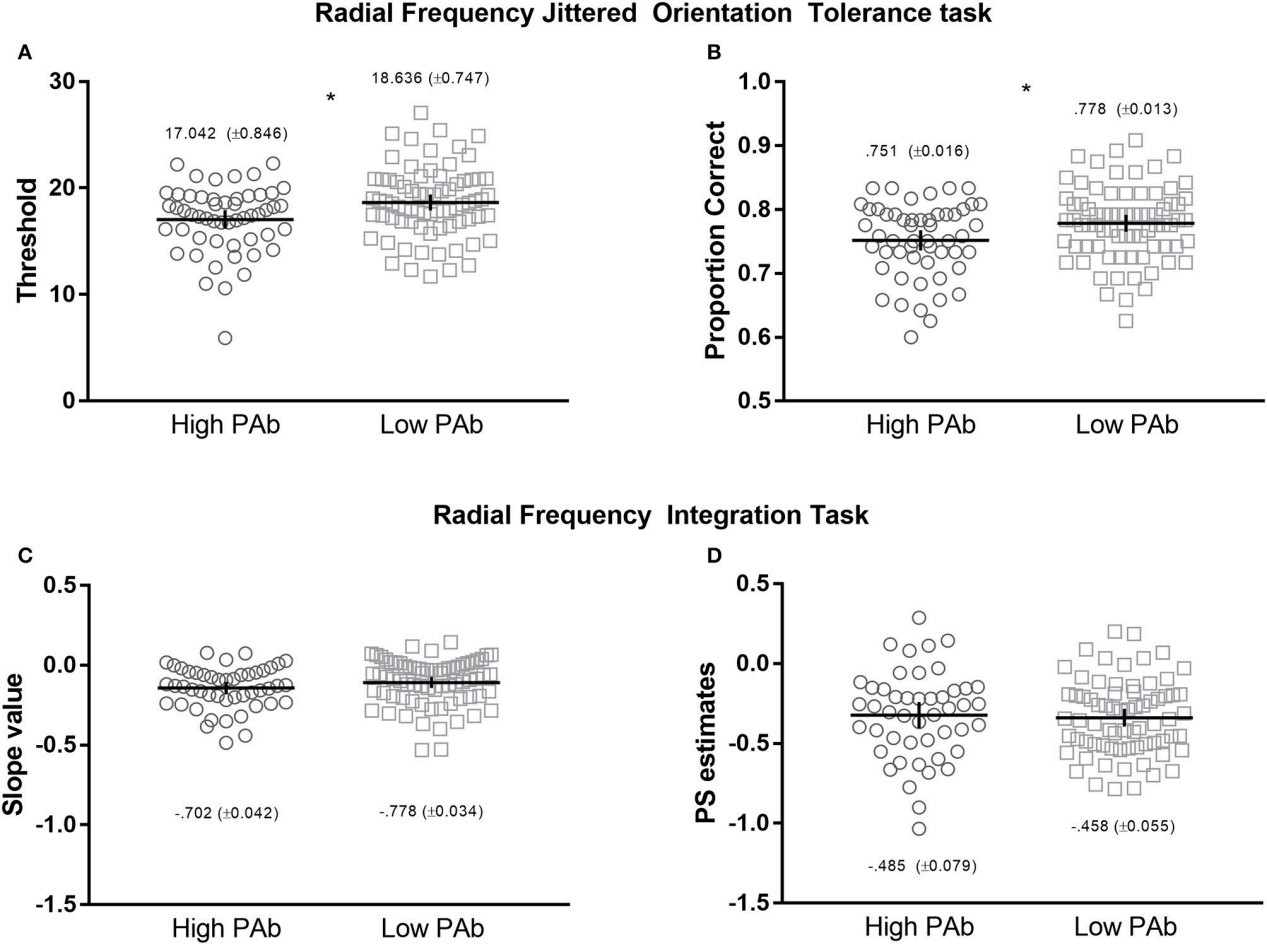
Upper: Radial Frequency Jittered Orientation Tolerance (RFJOT) task thresholds **(A)** and proportion of correct responses **(B)**—collapsed across jitter levels—between High vs. Low PAb. Lower: Radiacl Frequency Integration Task (RFIT) integration slope **(C)** and probability summation slope estimates **(D)** between High and Low PAb groups. ^*^p is significant at 0.05 level, error bars represent 95% CI.

Independent samples *t*-tests indicated that the High PAb group had statistically significantly lower orientation-noise thresholds than the Low PAb group, *t*_(126)_ = −2.744, *p* = 0.007, with a medium effect size, *d* = −0.494 (BF_10_ = 5.470^6^, Figure 2A), and a lower proportion of correct responses (collapsed across non-zero jitter levels; Figure 2B), *t*_(126)_ = −2.567, *p* = 0.011, *d* = −0.461, consistent with poorer local visual signal processing.

### RFIT

First, to determine whether global integration was present, separate repeated-measures ANOVA's were conducted for each group using the two slope values (integration slope and probability summation slope) as the dependent variables. A main effect of slope was observed for both the High PAb (*F*(1, 47) = 11.094), *p* = 0.001, η^2^ = 0.191) and Low PAb (*F*(1, 70) = 39.194), *p* < 0.001, η^2^ = 0.359) groups. For both comparisons, mean integration slope values were greater than the probability summation estimates (see Figure 2C,D), indicating that global integration occurred (32, 33, 49, 56).

Of note, the High PAb group had smaller mean integration slope values than the Low PAb group, however, the difference was not statistically significant, *t*_(117)_ = 0.517, *p* = 0.107, *d* = −0.302, which indicates that the efficiency of global integration did not systematically differ between these groups (Figures 2C,D).

### Additional analyses

Correlations between the key dependent measures of local and global visual integration were conducted to explore the potential overlap between tasks. A very small, non-significant correlation was found between RFIT integration slopes and RFJOT thresholds, *r*_(115)_ = 0.008, *p* = 0.930 and with RFJOT proportion correct, *r*_(119)_ = 0.038, *p* = 0.680, suggesting that the two tasks are measuring separate integration abilities, as expected.

Associations between other schizotypy traits (Magical Ideation, Social Anhedonia, Physical Anhedonia and Cognitive Disorganization) and visual integration outcomes were explored separately for (pooled) PAb groups (Table S3). A statistically significant correlation was found between Magical Ideation and RFJOT thresholds, with a small effect size, whilst correlations between negative schizotypy (Social Anhedonia and Physical Anhedonia) and disorganized (Cognitive Disorganization) traits and RFJOT/RFIT dependent measures were not statistically significant. These findings suggest that local contour integration abilities may be related to positive schizotypy traits, but not to negative or disorganized schizotypy.

## Discussion

Poor contour integration has been reported in chronic (14) and first episode patients with schizophrenia (18). The current results extend these earlier findings in two important ways. First, consistent with our predictions, the results show that poorer local processing of contour also occurs in high positive schizotypes in the general community. Specifically, the High PAb group displayed less tolerance to noise at the local signal level on the RFJOT task, indicating poorer integration of local orientation information than the Low PAb group. Second, and contrary to expectation, we found that global integration efficiency, assessed on the RFIT task, was intact in high positive schizotypes.

Since statistically significant PAb group differences on RFJOT threshold remained when AQ traits were added as a covariate, and both the negative and disorganization schizotypy traits were statistically non-significantly correlated with RFJOT thresholds, our findings point to poorer local contour processing being particularly associated with positive traits. Poorer contour integration in the High PAb group seems unlikely to be due to participants being unable to see or attend to the RFJOT stimuli, since there were no statistically significant differences between High and Low PAb groups in visual acuity or the proportion of correct responses when orientation jitter was absent.

The repeated finding of contour integration deficits across related paradigms [e.g., (14, 18, 22)] in both individuals vulnerable to, or diagnosed with, psychotic disorder [e.g., (18, 21)) suggests that abnormal contour integration may be a trait marker for schizophrenia. These findings are also consistent with Meehl's (6) concept of an integrative neural deficit (*schizotaxia*) underlying liability to this disorder. Importantly, however, the results of the current study suggest that poor tolerance to local orientation noise may provide a mechanistic basis of this neurointegrative deficit (57).

One implication of the current findings is that poorer local processing of contour in high schizotypes results in cascading negative effects, at later stages of information processing–such as difficulties with working memory (4). Could the direction of causality be reversed? That is, could a reduction in working memory in high schizotypy (58) lead to the difficulties in local noise tolerance observed in this paper? The current study is not designed to test this possibility. Since digit symbol coding performance draws on both the precision and capacity of working memory, yet did not differ in high and low schizotypes in the current sample, this line of argument seems unlikely. That said, digit symbol coding is clearly a multifactorial task, and not a specific index of components of working memory. Therefore, the possibility remains open that a selective difficulty in working memory precision alone, for example, could contribute to the poorer local contour processing observed in high schizotypes. Future work may look to alternative approaches to investigate this issue. For instance, tasks employing eye movement recording (59) may be useful to examine whether disruption in selective components of working memory in high and low schizotypes contributes to differences in local contour processing abilities.

The findings from this study in healthy adolescents and young adults suggest that positive traits are associated with poorer local integration throughout development (24), whereas global integration difficulties may only occur in those who progress to a diagnosis of psychosis. This proposal aligns with recent work in healthy observers which shows that local and global processing evolve independently across development (32). Using a variant of the RFIT, Cribb et al. (32) found that sensitivity to local curvature information continues to improve through childhood and adolescence, whilst global integration of shape information is already adult-like in early childhood. Given that most individuals with high levels of schizotypy do *not* go on to develop schizophrenia (60), we speculatively suggest that (i) the unusual perceptual experiences in high positive schizotypy are linked to poor local contour integration, and (ii) intact global integration of shape information in high schizotypes (as found in this study) may confer protection to the onset of psychotic disorder. Longitudinal study designs are now needed to examine this proposal. On the other hand, current models of schizophrenia propose that multiple genetic, environmental and cognitive risk factors contribute to the development of schizophrenia (61, 62). Identifying both similarities and differences between healthy schizotypes and individuals with schizophrenia will help to provide greater insight into the potential risk and protective factors for developing schizophrenia (3, 63).

Some additional limitations of this study should also be acknowledged. Firstly, the generalizability of the results is restricted by the confinement of participants to a university sample, given the selective nature of university entrance. Secondly, our study focused on positive schizotypy traits here, and have not experimentally controlled for levels of other schizotypy traits, which can be difficult given the high correlation between these traits (45). Therefore, conclusions regarding the specificity to positive traits needs to be tentative. On the other hand, our supplementary analyses showed that there were small, and often statistically non-significant correlations between other schizotypy traits (e.g., Social and Physical Anhedonia, and Cognitive Disorganization) and the key integration variables, highlighting the possibility of a specific relationship between positive schizotypy and atypical local integration. Nonetheless, the potential interaction amongst schizotypy traits is not fully understood (64), and should be a focus for future work.

In sum, this study demonstrates that healthy positive schizotypes have difficulty integrating local orientation information into coherent paths–similar to that previously found in schizophrenia patients (22). These findings suggest that impairments in local contour integration do not only occur after illness onset, they also characterize people at increased risk for psychosis.

## Ethics statement

The study protocol was approved by the Human Research Ethics Office at the University of Western Australia, and the study was carried out in accordance with their recommendations. All subjects provided informed written consent in accordance with the Declaration of Helsinki.

## Author contributions

KP, JB, JD, and DB contributed to designing the study. JD and DB created the stimuli and data collection programs. KP collected and analyzed the data. KP formed the manuscript in partial fulfillment of her Ph.D., with JB, JD and DB providing feedback. KP, JB, JD, and DB gave final approval and agreed to be accountable for all aspects of the work.

### Conflict of interest statement

The authors declare that the research was conducted in the absence of any commercial or financial relationships that could be construed as a potential conflict of interest.

## Footnotes

^1^We acknowledge that the RFIT can also measure sensitivity to local orientation signals [RFIT, threshold levels, for discussion see (26)], however, the primary purpose of the RFIT in this paper was as a measure of global integration

^2^Following the quasi-dimensional model of schizotypy underlying the Wisconsin Schizotypy Scales (39), the 90^th^ percentile was chosen as the cut-off to select the high schizotypy group, i.e., the upper 10% of schizotypes. Since “very low” scores may not be representative of the remaining population (40), the 50th percentile was chosen as the cut-off for selecting the low schizotypy group.

^3^The Psychosis Screen was used as a brief screen for the presence of psychosis. When 2+ (out of 6) items were screened as present, the Diagnostic Interview for Psychosis (a clinical interview for psychotic disorders) was used to provide a more detailed assessment.

^4^We utilized a slightly different method (further information in Supplementary Material) than previously used to obtain threshold estimates for the RFJOT, which had fewer free parameters, providing a reliable threshold estimate.

^5^A very small number of participants had psychometric function fits that indicated a relatively poor fit of the curve to their data. Analyses were repeated following the removal of participants where the *R*^2^ ≤ 0.6 for both the RFJOT (number removed: n_highPAb_ = 4, n_lowPAb_ = 3) and RFIT (number removed: n_highPAb_ = 3, n_lowPAb_ = 3).

^6^This Bayesian re-analysis was completed following the methods of Ly et al. (55) (please see Supplementary Results, Table S1 and Figure S1, for further details). This analysis also finds a moderate effect size in support of the hypothesis that the threshold is different between the High and Low PAb groups.

## References

[1] Silverstein SM. Visual perception disturbances in schizophrenia: a unified model. Neuropsychopathol Schizophr. (2016) 63:77–132. 10.1007/978-3-319-30596-7_4 27627825

[2] Silverstein SM Keane BP. Vision science and schizophrenia research: toward a re-view of the disorder editors' introduction to special section. Schizophr Bull. (2011) 37:681–9. 10.1093/schbul/sbr053 21700588 PMC3122283

[3] Etinger U Meyhöfer I Steffens M Wagner M Koutsouleris N Genetics, cognition, and neurobiology of schizotypal personality: a review of the overlap with schizophrenia. Front Psychiatr. (2014) 5:18 10.3389/fpsyt.2014.00018 PMC3931123 24600411

[4] Etinger U Mohr C Gooding DC Cohen AS Rapp A Haenschel C Cognition and brain function in schizotypy: a selective review. Schizophr Bull. (2015) 41:S417–26. 10.1093/schbul/sbu190 25810056 PMC4373634

[5] Debbané M Eliez S Badoud D Conus P Flückiger R Schultze-Lutter F. Developing psychosis and its risk states through the lens of schizotypy. Schizophr Bull. (2015) 41:S396–407. 10.1093/schbul/sbu176 25548386 PMC4373628

[6] Meehl PE Toward an integrated theory of schizotaxia, schizotypy, and schizophrenia. J Personality Disorders (1990) 4:1–99. 10.1521/pedi.1990.4.1.1

[7] de-Wit L Wagemans J Individual Differences in Local and Global Perceptual Organization. Oxford, UK: Oxford University Press (2015).

[8] Panton KR Badcock DR Badcock JC. A metaanalysis of perceptual organization in schizophrenia, schizotypy, and other high-risk groups based on variants of the embedded figures task. Front Psychol. (2016) 7:1–11. 10.3389/fpsyg.2016.00237 26941688 PMC4763090

[9] Silverstein SM Elliott CM Feusner JD Keane BP Mikkilineni D Hansen N. Comparison of visual perceptual organization in schizophrenia and body dysmorphic disorder. Psychiatr Res. (2015) 229:426–33. 10.1016/j.psychres.2015.05.107 26184989 PMC4546849

[10] Silverstein SM Keane BP. Perceptual organization impairment in schizophrenia and associated brain mechanisms: review of research from 2005 to 2010. Schizophr Bull. (2011) 37:690–9. 10.1093/schbul/sbr052 21700589 PMC3122298

[11] Uhlhaas PJ Silverstein SM. Perceptual organization in schizophrenia spectrum disorders: empirical research and theoretical implications. Psychol Bull. (2005) 131:618–32. 10.1037/0033-2909.131.4.618 16060805

[12] Dakin S Frith U. Vagaries of visual perception in autism. Neuron (2005) 48:497–507. 10.1016/j.neuron.2005.10.018 16269366

[13] Sun L Grützner C Bölte S Wibral M Tozman T Schlitt S. Impaired gamma-band activity during perceptual organization in adults with autism spectrum disorders: evidence for dysfunctional network activity in frontal-posterior cortices. J Neurosci. (2012) 32:9563–73. 10.1523/JNEUROSCI.1073-12.2012 22787042 PMC6622268

[14] Silverstein SM Keane BP Barch DM Carter CS Gold JM Kovács I. Optimization and Validation of a Visual Integration Test for Schizophrenia Research. Schizophr Bull. (2012) 38:125–34. 10.1093/schbul/sbr141 22021658 PMC3245579

[15] Butler PD Abeles IY Silverstein SM Dias EC Weiskopf NG Calderone DC. An event-related potential examination of contour integration deficits in schizophrenia. Front Psychol. (2013) 4:132. 10.3389/fpsyg.2013.00132 23519476 PMC3604636

[16] Feigenson KA Keane BP Roché MW Silverstein SM Contour integration impairment in schizophrenia and first episode psychosis: state or trait? Schizophr Res. (2014) 159:515–20. 10.1016/j.schres.2014.09.028 25306205 PMC4254521

[17] Keane BP Kastner S Paterno D Silverstein SM. Is 20/20 vision good enough? Visual acuity differences within the normal range predict contour element detection and integration. Psychonom Bull Rev. (2014) 22:121–7. 10.3758/s13423-014-0647-9 24845876 PMC4240750

[18] Keane BP Paterno D Kastner S Silverstein SM. Visual integration dysfunction in schizophrenia arises by the first psychotic episode and worsens with illness duration. J Abnormal Psychol. (2016) 125:543–9. 10.1037/abn0000157 27030995 PMC4850085

[19] Raine A The SPQ: a scale for the assessment of schizotypal personality based on DSM-III-R criteria. Schizophr Bull. (1991) 20:191–201. 10.1093/schbul/20.1.191 1805349

[20] Johnston MH Holzman PS Assessing Schizophrenic Thinking: A Clinical and Research Instrument for Measuring Thought Disorder. San Fransisco: Jossey-Bass (1979).

[21] Uhlhaas PJ Silverstein SM Phillips WA Lovell PG. Evidence for impaired visual context processing in schizotypy with thought disorder. Schizophr Res. (2004) 68:249–60. 10.1016/S0920-9964(03)00184-1 15099607

[22] Carter O Bennett D Nash T Arnold S Brown L Cai RY Sensory integration deficits support a dimensional view of psychosis and are not limited to schizophrenia. Transl Psychiatr. (2017) 7:e1118 10.1038/tp.2017.69 PMC5534945 28485725

[23] Grinter EJ Maybery MT Badcock DR. Vision in developmental disorders: is there a dorsal stream deficit? Brain Res Bull. (2010) 82:147–60. 10.1016/j.brainresbull.2010.02.016 20211706

[24] Tibber MS Anderson EJ Bobin T Carlin P Shergill SS Dakin SC. Local and global limits on visual processing in schizophrenia. PLoS ONE (2015) 10:e0117951. 10.1371/journal.pone.0117951 25689281 PMC4331538

[25] Wilkinson F Wilson HR Habak C. Detection and recognition of radial frequency patterns. Vis Res. (1998) 38:3555–68. 10.1016/S0042-6989(98)00039-X 9893789

[26] Almeida RA Dickinson JE Maybery MT Badcock JC Badcock DR. Enhanced global integration of closed contours in individuals with high levels of autistic-like traits. Vis Res. (2014) 103:109–15. 10.1016/j.visres.2014.08.015 25175114

[27] Bell J Badcock DR Wilson H Wilkinson F. Detection of shape in radial frequency contours: independence of local and global form information. Vis Res. (2007) 47:1518–22. 10.1016/j.visres.2007.01.006 17316737

[28] Dickinson JE Bell J Badcock DR. Near their thresholds for detection, shapes are discriminated by the angular separation of their corners. PLoS ONE (2013) 8:e66015. 10.1371/journal.pone.0066015 23741521 PMC3669261

[29] Dickinson JE Cribb SJ Riddell HR Badcock DR. Tolerance for local and global differences in the integration of shape information. J Vis. (2015) 15:1–24. 10.1167/15.3.21 25814547

[30] Dickinson JE McGinty J Webster KE Badcock DR. Further evidence that local cues to shape in RF patterns are integrated globally. J Vis. (2012) 12:1–17. 10.1167/12.12.16 23197768

[31] Tan KWS Bowden VK Dickinson JE Badcock DR. Modulated textures with shape structures implied by a closed flow are processed globally. J Vis. (2015) 15:1–18. 10.1167/15.3.17 25805177

[32] Cribb SJ Badcock JC Maybery MT Badcock DR. Dissociation of local and global contributions to detection of shape with age. J Exp Psychol Hum Percept Perform. (2016) 42:1761–9. 10.1037/xhp0000257 27379873

[33] Green RJ Dickinson JE Badcock DR Global processing of random-phase radial frequency patterns but not modulated lines. J Vis. (2017) 17:18–18. 10.1167/17.9.18 28837964

[34] Loffler G Wilson HR Wilkinson F. Local and global contributions to shape discrimination. Vis Res. (2003) 43:519–30. 10.1016/S0042-6989(02)00686-7 12594998

[35] Almeida RA Dickinson JE Maybery MT Badcock JC Badcock DR. Visual search targeting either local or global perceptual processes differs as a function of autistic-like traits in the typically developing population. J Autism Dev Disorders (2013) 42:1272–86. 10.1007/s10803-012-1669-7 23054202

[36] Winterstein BP Silvia PJ Kwapil TR Kaufman JC Reiter-Palmon R Wigert B Brief assessment of schizotypy: developing short forms of the Wisconsin Schizotypy Scales. Personality Individual Differences (2011) 51:920–4. 10.1007/s10862-011-9242-9

[37] Mason O Linney Y Claridge G. Short scales for measuring schizotypy. Schizophr Res. (2005) 78:293–6. 10.1016/j.schres.2005.06.020 16054803

[38] Chapman LJ Chapman JP Infrequency Scale. Unpublished Test (1983). From T. R. Kwapil, Department of Psychology, University of North Carolina at Greensboro, Greensboro, NC, 27402–6170.

[39] Grant P Green MJ Mason OJ. Models of schizotypy: the importance of conceptual clarity. Schizophr Bull. (2018). 44:1–8. 10.1093/schbul/sby012 29474661 PMC6188508

[40] Neill E. Methodological considerations in the recruitment and analysis of schizotypy samples. Front Psychiatr. (2014) 5:3. 10.3389/fpsyt.2014.00156 25414674 PMC4222126

[41] Castle D Jablensky A McGrath J Carr V Morgan V Waterreus A. The diagnostic interview for psychoses (DIP): development, reliability and applications. Psychol Med. (2006) 36:69–80. 10.1017/S0033291705005969 16194284

[42] Jablensky A McGrath J Herman H Castle D Gureje O Evans M. Psychotic disorders in urban areas: an overview of the study on low prevalence disorders. Aust N Z J Psychiatr. (2000) 34:221–36. 10.1080/j.1440-1614.2000.00728.x 10789527

[43] Fonseca-Pedrero E Ortuño-Sierra J Mason OJ Muñiz J The Oxford–liverpool inventory of feelings and experiences short version: further validation. Personality Individual Differences (2015) 86:338–43. 10.1016/j.paid.2015.06.041

[44] Fonseca-Pedrero E Palno M Ortuño-Sierra J Lemos-Giráldez S Muñiz J. Dimensionality of the Wisconsin Schizotypy Scales-brief forms in college students. Sci World J. (2013) 2013:1–8. 10.1155/2013/625247 24319377 PMC3844248

[45] Gross GM Silvia PJ Barrantes-Vidal N Kwapil TR. Psychometric properties and validity of short forms of the Wisconsin Schizotypy Scales in two large samples. Schizophr Res. (2012) 134:267–72. 10.1016/j.schres.2011.11.032 22189258

[46] Baron-Cohen S Wheelwright S Skinner R Martin J Clubley E. The Autism-Spectrum Quotient (AQ): evidence from asperger syndrome/high-functioning autism, males and females, scientists and mathematicians. J Autism Dev Disorders (2001) 31:5–17. 10.1023/A:1005653411471 11439754

[47] Dickinson JE Harman C Tan O Almeida RA Badcock DR. Local contextual interactions can result in global shape misperception. J Vis. (2012) 12:3. 10.1167/12.11.3 23035131

[48] Graham NVS Visual Pattern Analyzers. Oxford, UK: Oxford University Press (1989).

[49] Green RJ Dickinson JE Badcock DR Integration of shape information occurs around closed contours but not across them. J Vis. (2018) 18:6 10.1167/18.5.6 29904781

[50] Prins N Kingdom FAA Palamedes: Matlab Routines for Analyzing Psychophysical Data (2009). Available online at: http://www.palamedestoolbox.org/

[51] Oduntan OA Mashige KP Raliavhegwa-Makhado M A comparison of two methods of logMAR visual acuity data scoring for statistical analysis. South Afr Optometr. 63, 155–163. 10.4102/aveh.v68i3.162

[52] Atkins AS Davis VG Tseng T Vaughan A Harvey PB Narasimhan M Validation of the Tablet-based Brief Assessment of Cognition (BAC App) for Schizophrenia. (2014). Available online at: http://www.neurocogtrials.com/10.1016/j.schres.2016.10.010 27771201

[53] Cohen J Statistical Power Analysis for the Behavioral Sciences. New York, NY: Academic Press (1977).

[54] Crawford JR Garthwaite PH Ryan K. Comparing a single case to a control sample: testing for neuropsychological deficits and dissociations in the presence of covariates. Cortex (2011) 47:1166–78. 10.1016/j.cortex.2011.02.017 21458788

[55] Ly A Raj A Etz A Gronau QF Wagenmakers E-J Bayesian reanalyses from summary statistics: a guide for academic consumers. Adv Meth Pract Psychol Sci. (2018) 1:1–10. 10.1177/2515245918779348

[56] Tan KWS Dickinson JE Badcock DR. Detecting shape change: characterizing the interaction between texture-defined and contour-defined borders. J Vis. (2013) 13:12. 10.1167/13.14.12 24344055

[57] Winterer G Weinberger DR. Genes, dopamine and cortical signal-to-noise ratio in schizophrenia. Trends Neurosci. (2004) 27:683–90. 10.1016/j.tins.2004.08.002 15474169

[58] Xie W Cappiello M Park H-B Deldin P Chan RCK Zhang W. Schizotypy is associated with reduced mnemonic precision in visual working memory. Schizophr Res. (2018) 193:91–7. 10.1016/j.schres.2017.07.046 28760538

[59] Cappiello M Zhang W. A dual-trace model for visual sensory memory. J Exp Psychol Hum Percept Perform. (2016) 42:1903–22. 10.1037/xhp0000274 27786536

[60] Chapman LJ Chapman JP Kwapil TR Eckblad M Zinser MC. Putatively psychosis-prone subjects 10 years later. J Abnormal Psychol. (1994) 103:171. 10.1037/0021-843X.103.2.171 8040487

[61] Fusar-Poli P Bonoldi I Yung AR Borgwardt S Kempton MJ Valmaggia L. (2012). Predicting psychosis: meta-analysis of transition outcomes in individuals at high clinical risk. Arch Gen Psychiatr. 69:220–9. 10.1001/archgenpsychiatry.2011.1472 22393215

[62] Howes OD Murray RM. Schizophrenia: an integrated sociodevelopmental-cognitive model. Lancet (2014) 383:1677–87. 10.1016/S0140-6736(13)62036-X 24315522 PMC4127444

[63] Badcock JC Clark ML Pedruzzi RA Morgan VA Jablensky A. Intact speed of processing in a community-based sample of adults with high schizotypy: a marker of reduced psychosis risk? Psychiatr Res. (2015) 228:531–7. 10.1016/j.psychres.2015.06.003 26117248

[64] Debbané M Badoud D Balanzin D Eliez S. Broadly defined risk mental states during adolescence: disorganization mediates positive schizotypal expression. Schizophr Res. (2013) 147:153–6. 10.1016/j.schres.2013.03.012 23570898

