# Supplementary material for: Poorer Integration of Local Orientation Information Occurs in Students With High Schizotypal Personality Traits
Source: Front Psychiatry. 2018 Oct 24;9:518. doi: 10.3389/fpsyt.2018.00518 (PMC6207847; doi:10.3389/fpsyt.2018.00518)
Supplement: Supplementary file 1 [file Data_Sheet_1.pdf]

1. Supplementary methods

1.1 Materials

The RFIT and RFJOT stimuli were created in MatLab 7.0.4 (Mathworks, Natick, MA, USA), through a Pentium 4, 3GHz PC. An Optical OP 200-E photometer (head model number 265) was used to calibrate the screen. The background luminance was set to 45 cd/m^2, maximum luminance of 90 cd/m^2 and the screen resolution was 1024 x 768 pixels. The stimuli were displayed on a Sony Triniton monitor (100Hz refresh rate) via the frame buffer of a Cambridge Research Systems (CRS) ViSaGe (CRS, Kent, UK) visual stimulus generator. A chin-rest was used to maintain a distance of 65.5cm from the screen, which produces pixels subtending 2’ of visual angle. A CRS CB6 button-box was used to make responses.

1.2 RFJOT analysis

Data were collected for a range of different orientation jitter amounts. Proportion correct (out of 20) for each jitter level was extracted, and a curve fitted describing the change in proportion correct as a function of jitter amount using:

$$Proportion\ correct = 1 - (.5.\left(.5.\left(1 + error\ function(\frac{Jitter - Threshold}{\sigma.\sqrt{2}})\right)\right)) \quad \text{(S1)}$$

For each individual, our primary measure, *threshold, was* derived by fitting Equation S1 to the data. The threshold represents the amount of jitter that causes performance to decline to 75%, such that a lower threshold signifies poorer integration, that is, a lower amount of jitter was tolerated.

In addition, a second analysis calculated the total number of trials in which the answer was correct so this could also be compared across groups, as has been the practice with the JOVI task (Silverstein et al., 2012).

1.3 RFIT analysis

To determine an index of integration performance (our primary measure of performance for this task), a power function was fit to the thresholds for each number of cycles:

$$Threshold = k\,(number\ of\ cycles)^{B} \qquad \text{(S2)}$$

where *k* is a sensitivity scaler, and *B* is the rate of change in threshold as number of cycles is increased (from here on referred to as *slope*). To determine whether global integration has occurred, the slope was compared to *probability summation.* If global integration has occurred, the integration slope will be significantly steeper than the PS slope. Signal detection theory (SDT) was used to generate probability summation estimates (PS). d prime (*d'*) was determined by:

$$d' = (gA)^{\tau} \qquad \text{(S3)}$$

such that *d'* is the internal strength of a signal, *g* is a scaling factor which includes the reciprocal of the internal noise standard deviation, *A* is the stimulus intensity, and τ is the internal transducer (that is, the rate at which the observer is converting increased stimulus intensity to increased perceptual salience). The percentage correct for PS estimate using SDT was computed by:

$$PC = n\int_{-\infty}^{\infty}\phi(t-d')\Phi(t)^{QM-n}\Phi(t-d')^{n-1}dt + (C-n)\ ...$$

$$\int_{-\infty}^{\infty}\phi(t)\Phi(t)^{QM-n-1}\,\Phi(t-d')^{n}dt \qquad \text{(S4)}$$

where PC is the percentage correct (set at 75%), *t* is sample stimulus strength, the heights of the noise and signal distributions at *t* are given by $\phi(t)$ and $\phi(t - d')$ respectively, $\Phi(t)$ and $\Phi(t - d')$ are the areas under the noise and signal distributions to the left of *t*, *C* is the number of monitored channels, *M* is the number of alternatives in the forced choice task, and *n* is the

number of stimulus components. This equation was executed through the Palamedes toolbox (Prins & Kingdom, 2009)[1]. For a proportion of individuals (n = 12[2]) reliable slope estimates were unable to be calculated, and therefore were excluded from analyses reported in the paper.

2. Supplementary results

The data of individual participants were usually well fitted by a cumulative Gaussian function (Equation S1) and R^2 values are reported in the manuscript indicating that fit quality. Under those circumstances it is common to average the parameters of the curve fits (mean and sigma) when producing a group plot because individual curves can vary in both slope and position along the signal intensity axis. An alternative method of producing group plots is to average the performance at each signal intensity level and then fit a cumulative Gaussian function to that averaged data. This produces a slightly different threshold estimate for the group and a shallower curve, since averaging steeper curves in different positions tends to blur them. In this study, each approach finds significant group differences between the threshold estimates so the outcome of the study is not influenced by this choice. However, for information, we present the group psychometric functions, graphically to show the outcomes for the two methods. See Figures S1A (the curves arising from the averaged curve fit parameters) and S1B (the curves arising from the averaged raw data at each orientation jitter level followed by a single curve fit for each group). The estimated parameters are also provided in Table S1 which shows the increase in sigma, which influences the slope of the curve fits. However, since in each case the

[1] available at http://www.palamedestoolbox.org
[2] PAb group: Low, n = 6 (7.79%); High, n = 7 (12.73%)

High PAb group has a significantly lower threshold estimate than the Low PAb group, the interpretation remains the same.

A Bayesian reanalysis of our data was also conducted to investigate the strength of the evidence for the hypothesis that the group means were different. This analysis, following the methods recommended in Ly, Raj, Etz, Gronau, and Wagenmakers (2018) produced the Bayes factor, BF_{10}, from our *t*-test results, using JASP (version 0.9.0.1, JASP Team, 2018) with the default prior, Cauchy scale value 0.707 and the Bayes factor robustness check. The Bayes factor indicates the strength of support for the hypothesis that the groups are different relative to the hypothesis that they are the same.

When performing this calculation with the analysis presented in the main document, where individually fitted curve parameters are compared across groups (Figure S1A), the threshold difference produces a BF_{10} of 5.47, which is regarded as “moderate” evidence, and indicating the hypothesis that the groups are different is 5.47 times more likely than the hypothesis that they are the same. This corresponds with the conclusion of a moderate effect size in the main analysis. The robustness check does show that the BF_{10} value would vary with the Cauchy scale value, which alters the width of the assumed prior distribution, but all BF_{10}s produced with values greater than 0.05 stay within the “moderate” range, so the conclusion does not depend on this choice.

An identical analysis based on the aggregated jitter orientation level data, followed by a single curve fit for each group (see Figure S1B) produces a BF_{10} = 62.03 (assuming Cauchy 0.707), indicating “very strong” evidence in support of the hypothesis that the average group thresholds are different. Again the robustness check shows a variation in BF_{10} values when the Cauchy scale value is varied but the values remain in the “very strong” range. We do not prefer

plotting the data this way because it produces shallower slope estimates on the fitted curves (see Figure S1C for a direct comparison) but these additional analyses show that our conclusions are unaffected by this choice.

Table S1. Comparison of Gaussian parameters (threshold and sigma) for averaged individual fits (left) and aggregated raw data for each orientation jitter level fit for High and Low schizotypy groups (right).

	Averaged individual fits (as in the paper)		Aggregated raw data fit	
	High PAb	Low PAb	High PAb	Low PAb
Threshold	17.042 (± 0.846)	18.635 (± 0.747)	17.060 (± 0.514)	18.300 (± 0.430)
Sigma	5.297 (± 0.923)	6.006 (± 0.746)	6.364 (± 0.745)	6.841 (± 0.638)
R^2	0.860	0.886	0.743	0.738

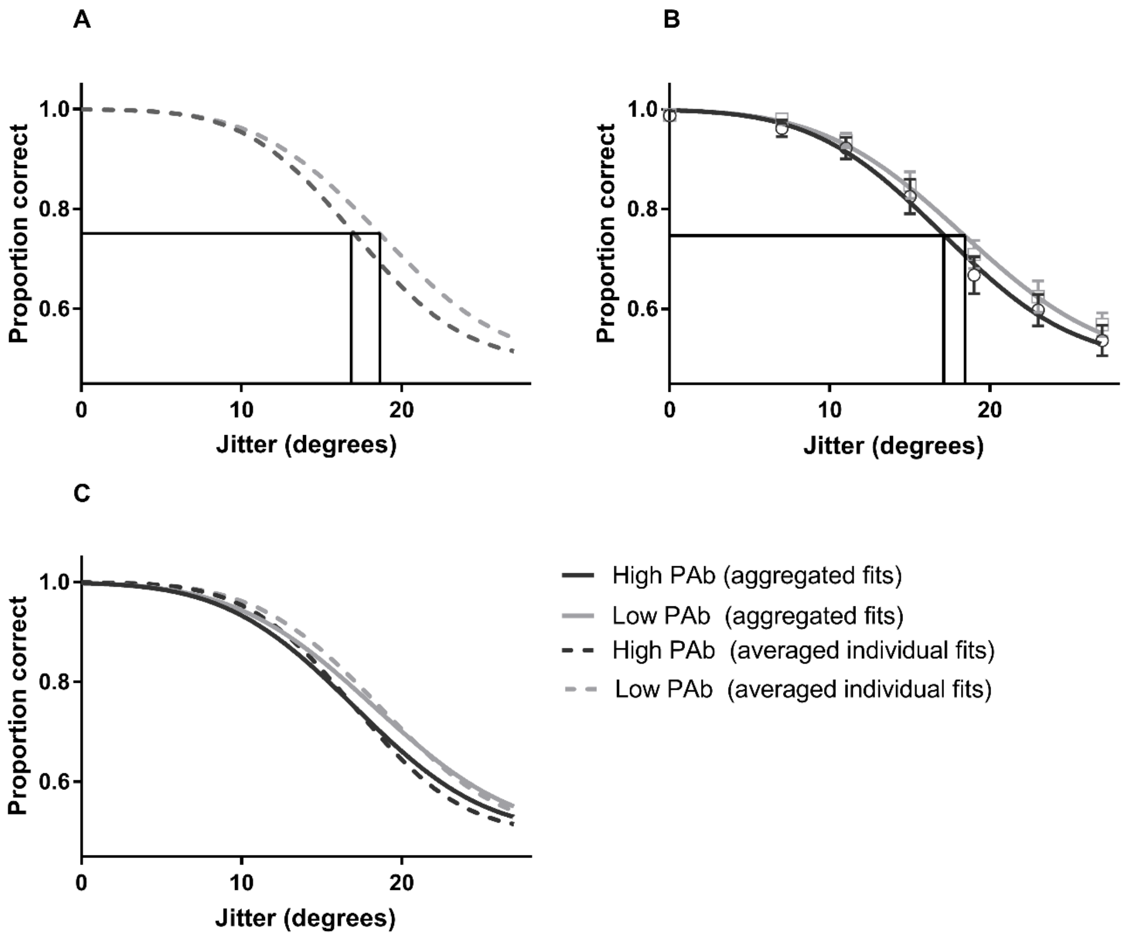

Figure S1. The left graph (A) represents a simulated curve using the data from the paper, where individual fits were averaged for High and Low PAb groups. The centre graph displays the aggregated fits for High and Low PAb groups. The right graph (C) shows how each fit provides a different representation of the data. 75% threshold is extracted from this fit (solid black line).

Table S2. Perceptual Aberration scores for schizotypy samples (all university/college samples) across different studies.

	N	*M* (*PAb*)	*SD* (*PAb*)	*Range* (*PAb*)	*% Males*	*M* (*Age*)
Current screening sample	1686	1.04	2.07	0-15	34.5*	20.18*
Winterstein et al. (2011)	1144	1.32	2.27	0-15	24.0	22.90
Fonseca-Pedrero, Palno, Ortuño-Sierra, Lemos-Giráldez, and Muñiz (2013)	1349	1.12	1.88	0-13	21.1	20.48
Gross, Silvia, Barrantes-Vidal, and Kwapil (2015)	2292	1.29	2.12	0-15	24.0	19.50
Gross, Silvia, Barrantes-Vidal, and Kwapil (2012)	6137	1.22	2.29	0-15	24.0	19.40

**Note:* 5.6% of the sample did not specify a gender or age; PAb = Perceptual Aberration Scale.

Table S3. Correlation of schizotypy traits and participant characteristics with visual integration measures across pooled PAb groups.

	RFJOT threshold (*n* = 128)	RFJOT PC (*n* = 128)	RFIT slope estimate (*n* = 118)	RFIT PS estimate (*n* = 118)
Schizotypy traits				
Magical Ideation	-.203*	-.170	-.084	.094
Social Anhedonia	.022	.033	.112	.034
Physical Anhedonia	-.069	-.063	.040	.052
Cognitive Disorganisation^	-.172	-.153	-.001	.064
Autism Quotient	-.153	-.095	-.058	-.029
Digit Symbol Coding^	-.027	-.025	-.180*	.025
Acuity^	-.171	-.121	-.055	.021
Handedness	-.079	-.104	.070	-.058

Note: **p* is significant at .05 level (two-tailed); ^ Pearson's correlations.

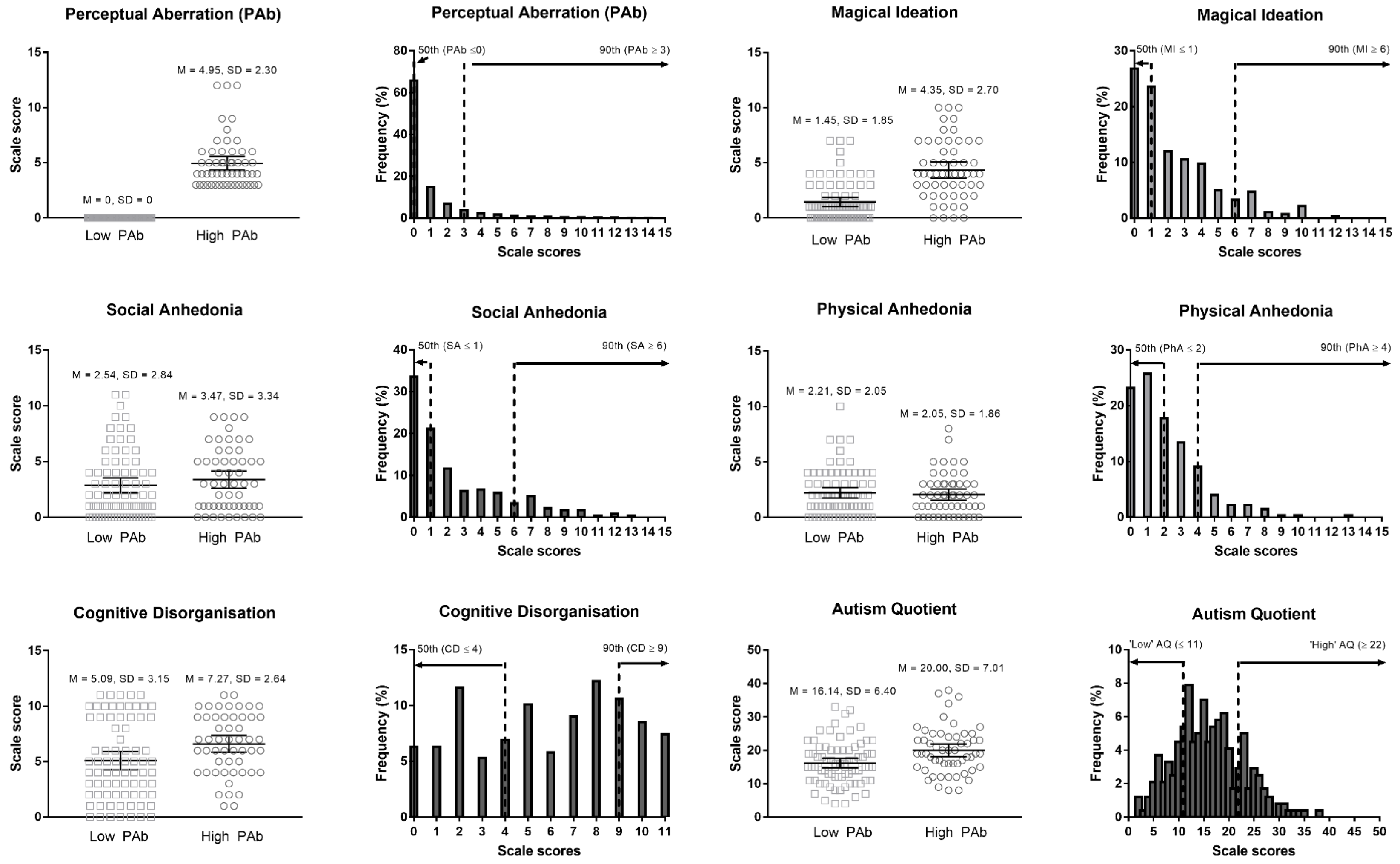

Figure S2. Columns 1 and 3 represent the spread of schizotypy and AQ scores between High and Low PAb groups. Columns 2 and 4 show the frequency distribution of these traits using re-test scores (apart from the PAb, where screening scores were used). The 50th and 90th percentile cut-offs are indicated by the dotted lines on columns 2 and 4 (aside from the AQ, where a cut-off from a previous study was used, Almeida, Dickinson, Maybery, Badcock, & Badcock, 2010)). *Note:* error bars represent 95% CI.

References

Almeida, R. A., Dickinson, J. E., Maybery, M. T., Badcock, J. C., & Badcock, D. R. (2010). A new step towards understanding Embedded Figures Test performance in the autism spectrum: the radial frequency search task. *Neuropsychologia, 48,* 374-381. doi:10.1016/j.neuropsychologia.2009.09.024

Fonseca-Pedrero, E., Palno, M., Ortuño-Sierra, J., Lemos-Giráldez, S., & Muñiz, J. (2013). Dimensionality of the Wisconsin Schizotypy Scales-Brief Forms in College Students. *The Scientific World Journal, 2013,* 1-8. doi:http://dx.doi.org/10.1155/2013/625247

Gross, G. M., Silvia, P. J., Barrantes-Vidal, N., & Kwapil, T. R. (2012). Psychometric properties and validity of short forms of the Wisconsin Schizotypy Scales in two large samples. *Schizophrenia Research, 134*(2), 267-272.

Gross, G. M., Silvia, P. J., Barrantes-Vidal, N., & Kwapil, T. R. (2015). The dimensional structure of short forms of the Wisonsin Schizotypy Scales. *Schizophrenia Research, 166*(1), 80-85. doi:http://dx.doi.org/10.1016/j.schres.2015.05.016

JASP Team. (2018). JASP (Version 0.9)[Computer Software]. Retrieved from https://jasp-stats.org/

Ly, A., Raj, A., Etz, A., Gronau, Q. F., & Wagenmakers, E.-J. (2018). Bayesian Reanalyses from Summary Statistics: A Guide for Academic Consumers. *Advances in Methods and Practices in Psychological Science, 1*(3). doi:10.1177/2515245918779348

Prins, N., & Kingdom, F. A. A. (2009). Palamedes: Matlab routines for analyzing psychophysical data., Retrieved from: http://www.palamedestoolbox.org/.

Silverstein, S. M., Keane, B. P., Barch, D. M., Carter, C. S., Gold, J. M., Kovács, I., . . . Strauss, M. (2012). Optimization and Validation of a Visual Integration Test for Schizophrenia Research. *Schizophrenia bulletin, 38*(1), 125-134. doi:10.1093/schbul/sbr141

Winterstein, B. P., Silvia, P. J., Kwapil, T. R., Kaufman, J. C., Reiter-Palmon, R., & Wigert, B. (2011). Brief assessment of schizotypy: Developing short forms of the Wisconsin Schizotypy Scales. . *Personality and Individual Differences, 51*, 920-924. doi:10.1007/s10862-011-9242-9
